# Targeting neuroglial aromatase for CNS injuries: molecular insights and therapeutic prospects

**DOI:** 10.1186/s13041-026-01342-x

**Published:** 2026-08-19

**Authors:** Min Zhu, Qi Wang, Rongjia Li, Likun Yang, Maohu Zhou, Xuan Zhang, Chuang Zhou, Xiao Zhao, Jiqiang Zhang, Zhaoyou Meng

**Affiliations:** 1 Department of Nursing, the 904th Hospital of PLA Joint Logistics Support Force, Wuxi, 214000 China; 2 Department of Neurobiology, Army Medical University, Chongqing, 400038 China; 3 Department of Neurosurgery, the 904th Hospital of PLA Joint Logistics Support Force, Wuxi, 214000 China; 4 Department of Neurology, Xinqiao Hospital, Army Medical University, Chongqing, 400037 China

**Keywords:** Aromatase, Neuroglia, Neuroprotection, Sexual dimorphism, Brain injury, Estrogen signaling

## Abstract

Aromatase (AROM) is the key enzyme that catalyzes estrogens (mainly 17β-estradiol, E2) biosynthesis. While high levels of AROM have been detected in specific neurons in the central nervous system (CNS), emerging evidence reveals differential levels of AROM in nonneuronal cells such as astrocytes, microglia, and ependymal cells. This review integrates current knowledge on the dynamic regulation and neuroprotective roles of neuroglial AROM, particularly following CNS injuries such as traumatic brain injury and ischemic stroke. Neuroglia cells, especially astrocytes, significantly fluctuate in AROM expression in response to injury and disease, which is mediated by E2 feedback and cytokine signaling. Astrocyte-derived E2 exerts multiple neuroprotective effects through genomic (ERα/ERβ activation) and nongenomic pathways (GPR30-mediated signaling), modulating neuroinflammation by regulating microglial activation and reducing reactive astrogliosis. Notably, sexual dimorphism in AROM regulation, with females exhibiting higher basal and injury-induced AROM activity, may explain their greater resilience to brain injuries. Although challenges remain in optimizing blood‒brain barrier penetration and sex-specific dosing regimens, targeting neuroglial AROM and its downstream effectors exhibits promise for enhancing postinjury recovery. Future studies should employ multiple omics methods to characterize AROM-positive glial subpopulations and develop conditional knockout models to dissect cell type-specific mechanisms, ultimately paving the way for precision therapies for the treatment of brain injuries, AROM-associated neural disease and other CNS disorders.

## Introduction

Aromatase (AROM, encoded by the *Cyp19A1* gene) belongs to the cytochrome P450 family 19 subfamily A. This enzyme functions as an estrogen synthase, catalyzing androgen into estrogens (especially 17β-estradiol, E2), presents in various tissues, such as female [1–5] and male [6, 7] reproductive organs, bone and fatty tissue [8], skin [9, 10], liver [11], and blood vessels [12, 13]. Notably, extragonadal synthesis is the predominant source of E2 after menopause in women, and many previous publications have investigated the potential roles of local AROM in the regulation of various physiological and pathological conditions[14–19] via the use of AROM inhibitors[20–25] and/or AROM knockdown/knockout animals/cells[8, 13, 26–32]. The effect of E2 is mediated by two kinds of estrogen receptors (ERs): the classical nuclear/nonnuclear receptors (ERα and ERβ), and G-protein-coupled receptor 30 (GPR30, also named GPER1) in the plasma membrane and in the endoplasmic reticulum[33] which may not be a direct ER as stated by a recent study [34].

In the central nervous system (CNS), AROM expression is predominantly detected in neurons [35–42], but it is also detectable in neuroglial cells at different levels, at specific stages of development and under specific conditions [37, 38, 41–44]. At the subcellular level, AROM is predominantly localized in the neuronal endoplasmic reticulum [45–47], synaptic terminals [43, 48], pre- and postsynaptic components, as evidenced by immunoGold electron microscopy [37], or in the cytoplasm of astrocytes [49]. A recent “complex” fluorescent immunocytochemical analysis revealed that in the rat brain, AROM was localized in the nucleus of astrocytes but in the cytoplasm of microglia [41].

One of the most fascinating findings is the discovery of brain AROM by Naftolin and colleagues [50–53]. These pioneering studies laid the foundation for the brain AROM hypothesis, as reviewed by Lephart ED [54]. Many studies in this field have linked brain AROM with neuroendocrine events and behaviors, such as synaptic plasticity [33, 55–57], neurogenesis [58, 59], neuro-responses to injury [29, 60–62], mood [63, 64], cognition [44, 65, 66], learning and memory [66–69] and neurodegenerative diseases (NDDs), such as Alzheimer’s disease (AD) [69–73]. Here, we integrate the current progress in understanding the expression and function of neuroglial cells, highlighting their therapeutic potential in response to brain injuries and other neural disorders.

## AROM is differentially expressed in neuroglial cells

Besides neuronal localization, central AROM is also detectable in neuroglial cells at different levels, at specific developmental stages and under specific conditions.

### AROM expression in astrocytes

Early studies using cultured cells from adult male rats [74, 75] and perinatal hypothalamic cells from mice [40, 76] reported that neurons are the only AROM-expressing cells. Later studies reported lower AROM activities in astrocytes from rat embryos [36], from the neonatal cortex (in which AROM was inhibited by interleukin-1 in a dose-dependent manner) [49], from adult hippocampal slices [37] and from bovines and dogs [38, 77]. Recent studies have shown that in the migrating ventricular-subventricular zone (V-SVZ), AROM expression has been detected in astrocytes surrounding migrating neuroblasts [78] as evidenced with fluorescence immunocytochemistry [41] and fluorescence in situ hybridization [42]; AROM expression has also been detected in a rat glioblastoma C6 line, human glioblastoma cell lines (T98G and U373MG) [79], human AD patients and healthy controls [43], and the epileptic hippocampus [80]. Interestingly, in the prefrontal cortex (PFC) of individuals with AD, astrocytic AROM was increased in the late stages [81].

### AROM expression in microglia

Only two studies have reported the expression of AROM in microglia. The first study revealed that in the adult rat brain, both ramified and amoeboid microglia expressed AROM [41]. The second study revealed that in female multiple sclerosis brain samples, AROM expression was detected in isolated microglia and not regulated by TNF, whereas in males, AROM levels were positively correlated with TNF-α levels in primary cultures of human microglia. These differences may contribute to the sex differences observed in the prevalence of multiple sclerosis [82].

### AROM expression in oligodendrocytes

Oligodendrocytes seem to be completely negative for AROM in cultured 14–15-day-old rat embryos [36, 39] and neonatal rat pups [83, 84]. However, in oligodendrocytes in the hippocampal slices of adult rats [37], human AD patients and healthy controls [43], lower levels of AROM were detected. Therefore, it seems that at early developmental stages, oligodendrocytes do not express AROM; however, at later stages (even in adults), AROM is detectable in these cells, albeit at a lower level.

### AROM expression in ependymal cells and radial glial cells

In the dog brain [38] and human brain [43], AROM immunoreactivity (AROM-ir) was detected in ependymal cells. In the pejerrey fish brain, it was strongly detected in ependymal cells in a male-predominant manner [85]. Interestingly, AROM-ir was clearly diminished in the ependymal (and choroid plexus cells) lining of the lateral and third ventricles in the AD brain compared with that in control patients [43].

The CNS of adult teleost fish is characterized by the persistence of radial glial cells and very high levels of AROM [86, 87]. In the rainbow trout brain, very high levels of AROM were detected in radial glial cells, and a weaker signal was detected in the ependymal layer [88]; in female African cichlid fish, in situ hybridization revealed AROM expression in radial glial cells [89].

**Fig. 1:**
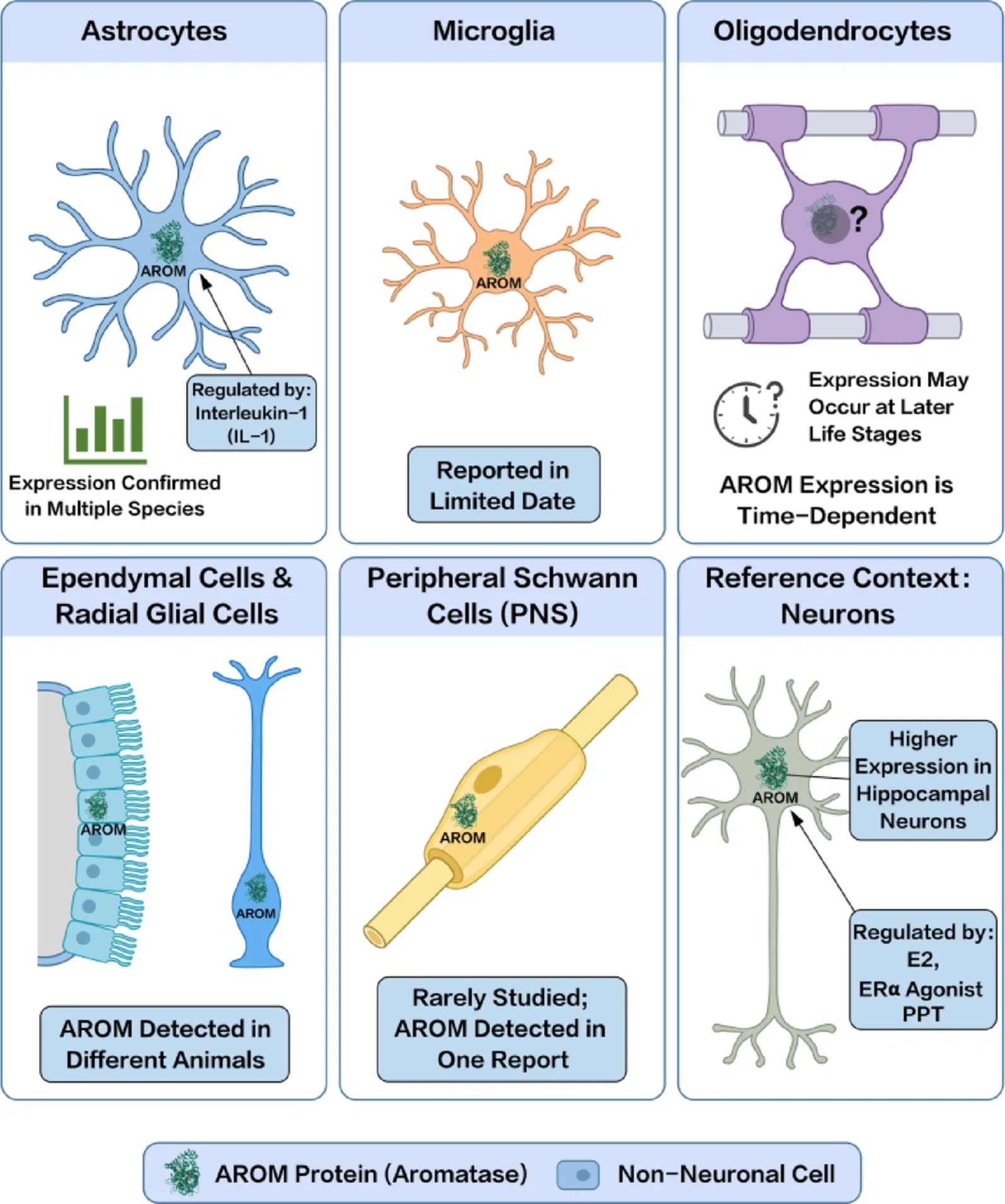
Schematic overview of aromatase (AROM) expression in various nonneuronal cells, with the neuronal context for reference

Taking together, AROM expression has been detected in nonneuronal cells of several species, with lower levels than those detected in neurons. Additionally, one report revealed that AROM-ir was detected in the cytoplasm of Schwann cells [90]. Glial AROM expression pattern showed several features. The first is developmental dynamics. For example, oligodendrocytes show stage-dependent expression, negative in early development but detectable at low levels in adults; teleost fish retain high AROM in radial glia throughout adulthood. The second is that the neuroglial expression seems to be inducible, astrocytic AROM increases in response to injuries and diseases such as epilepsy and AD. The third is methodological variations (e.g., enzyme activity detection, immunocytochemistry *vs.* in situ hybridization) and species-dependent (e.g., mammalian *vs* teleost). The last is sex-dependent pattern, such as male microglial AROM correlates with TNF-α. These findings are summarized in Table 1 and Fig. 1.

**Table 1:** AROM expression in neuroglial cells

Cell type	Experimental conditions/species	Key findings	References
Astrocytes	Rat embryos	Lower AROM activity detected	[36]
	Neonatal rat cortex	AROM inhibited by interleukin-1 (dose-dependent)	[49]
	Adult rat hippocampal slices	Detectable AROM expression	[37]
	Bovine/dog brains	Lower AROM levels	[38, 77]
	Rat V-SVZ (developing brain)	Expressed in astrocytes surrounding migrating neuroblasts	[78]
	Human AD patients & controls	Increased in late-stage AD prefrontal cortex; detected via immunocytochemistry	[43, 81]
	Rat glioblastoma (C6), human glioblastoma (T98G, U373MG)	Detectable expression	[79]
	PFC in AD and epileptic hippocampus	Detectable expression	[43, 80, 81]
Microglia	Adult rat brain	Expressed in ramified and amoeboid microglia	[41]
	Human multiple sclerosis	Detected in isolated microglia; sex-specific regulation (positive correlation with TNF-α in males only)	[82]
Oligodendrocytes	Cultured rat embryos (E14-15)	No detectable expression	[36, 39]
	Neonatal rat pups	No detectable expression	[83, 84]
	Adult rat hippocampal slices	Low levels detected	[37]
	Human AD patients & controls	Low levels detected	[43]
Ependymal cells	Dog brain	AROM-ir detected	[38]
	Human brain	AROM-ir detected; diminished in AD lateral/third ventricles	[43]
	Pejerrey fish brain	Strong male-predominant expression	[85]
	Rainbow trout brain	Weak expression	[88]
Radial glial cells	Adult teleost fish CNS	Very high levels	[86, 87]
	Rainbow trout brain	Very high levels	[88]
	Female African cichlid fish	Detected via in situ hybridization	[89]
Schwann cells	Peripheral nervous system	Cytoplasmic AROM-ir	[90]

## Functions, mechanisms and sex differences of neuroglial AROM

Accumulated studies have reported that neuroglial AROM can be induced by various stimuli, such as E2 treatment [91], canine distemper virus (CDV) infection [92]; while 1,25-dihydroxyvitamin D(3) stimulates AROM in human glioma but not in human neuroblastoma cells or rat astrocytes [93]. Importantly, glial AROM can be significantly induced by brain injuries and specific chemical and physical stimuli.

### Astrocytic AROM in neurogenesis

During neurogenesis, neuroblasts in the V-SVZ migrate along the rostral migratory stream into the olfactory bulb in the tube-like structure formed by astrocytes. AROM expression is detected in astrocytes surrounding migrating neuroblasts, suggesting that astrocytes play a protective role by producing E2, which may be mediated by GPR30, since the inhibition of GPR30 signaling leads to an increase in the p-cofilin/cofilin ratio, an indicator of actin cytoskeletal homeostasis [78].

### Astrocytic AROM in neural diseases

*Epilepsy*. This is a long-term/chronic disease that causes repeated seizures due to abnormal electrical signals produced by damaged brain cells. Kainic acid (KA), an acidic pyrolidine isolated from the seaweed Digenea simplex, is the most commonly used exogenous excitotoxin for the establishment of an experimental epilepsy model [94]. In both mice and rats, KA treatment did not affect neuronal AROM expression, but it induced dramatic AROM expression and activity in hippocampal reactive astrocytes, followed by other brain areas that are affected by KA, such as in the astrocytes of the entorhinal cortex, amygdaloid complex, pyriform and bed nuclei of the stria terminalis [95]. Notably, in the normal and epileptic human hippocampus, a small subpopulation of astrocytes presented AROM-ir [80]. Another study showed that in KA-induced rats, systemic or intrahippocampal AROM inhibitor treatment after seizure onset strongly suppresses both electrographic and behavioral seizures [96], indicating the potential role of AROM inhibitors in the treatment of human epilepsy [97]. However, it is noteworthy that while ARTOM inhibitors treatment reduces seizures, they may also exacerbate neurodegeneration in epileptic animal models, suggesting a great therapeutic challenge for clinical application [35, 98].

*Multiple sclerosis*. Multiple sclerosis is a chronic autoimmune neurodegenerative condition that causes demyelination and axonal damage. In the white matter of human multiple sclerosis patients, increased expression of AORM and ERβ was detected only in males, indicating that AROM may contribute to sex differences and the prevalence and course of multiple sclerosis [82]. The latest study showed that in the spinal cord of experimental autoimmune encephalomyelitis (EAE) mice, a rodent model of multiple sclerosis, the positive rate in glial cells and AROM-ir rates decreased after EAE induction, whereas the ERα and ERβ immunopositivity rates increased; naringenin (an estrogenically effective flavonoid) improved AROM-ir rates and gene expression in both prophylactic and therapeutic use, although it could not prevent or eliminate EAE lesions completely [99]. These results indicate the complex role of astrocytic AROM in multiple sclerosis, and further experimental evidence is urgently needed.

### Astrocytic AROM in brain injury

Astrocytic AROM can be profoundly regulated by brain injuries such as acquired brain injury (ABIs; including trauma, strokes, infections, tumors, or lack of oxygen) caused by traumatic brain injury (TBI) and non-TBI, which affects more than 85 million people globally each year and results in disorders in memory, mobility, emotions, and independence [100].

First, astrocytic AROM can be induced by TBI, and its inhibition may increase the risk of neurodegeneration [101]. When increased pressure (25 mm Hg) was used to mimic TBI, cultured rat primary cortical astrocytes presented significant increases in both AROM mRNA and protein levels, indicating that astrocytes can sense increased pressure after TBI and increase AROM production in response to this injury [102]. In a controlled cortical impact (CCI) model of TBI, treatment with the AROM inhibitor letrozole (LET) exacerbated neurological deficits and increased the number of reactive astrocytes, specifically in females [103].

Many studies explored the role of glial AROM in ischemic brain injury, including ischemic stroke, global cerebral ischemia (GCI) and ischemia‒reperfusion injury, which induces complicated neuroimmune inflammatory responses that significantly influence disease progression and patient outcomes [104]. Carswell et al. provided the first evidence that in experimental stroke (middle cerebral artery occlusion; MCAO) animals, increased astrocytic AROM expression was detected in the penumbra/peri-infarct zones post-MCAO, indicating the potential for astrocytic AROM to promote the survival of cells in the penumbra zone [105]. In adult GCI rats, AROM was highly expressed in astrocytes in the hippocampal CA1 region; antisense oligonucleotides against AROM resulted in a significant increase in GCI-induced neuronal cell death and neuroinflammation, indicating that astrocytic AROM is neuroprotective and anti-inflammatory following GCI [106]. As evidence, in GFAP promoter-driven aromatase knockout (GFAP-ARO-KO) mice, GCI did not induce the increase in astrocytic AROM, whereas increased neuronal damage and microglial activation as well as cognitive deficits were observed. RNA sequencing revealed that the hippocampus of ischemic GFAP-ARO-KO mice failed to upregulate A2-reactive astrocyte genes and that the JAK-STAT3 pathway was significantly downregulated following GCI [29]. A subsequent tamoxifen-inducible astrocyte-specific AROM knockout mouse line (GFAP-ARO-iKO) revealed that after GCI, significant hippocampal reactive astrogliosis, increased neuronal loss and microglial activation were observed [30]. The above effects may be, at least in part, mediated by GPR30, since its agonist G1 significantly prevents reactive astrocyte loss, increases the expression of STAT in CA1 astrocytes and hippocampal neurogenesis, enhances the expression of synaptic proteins and cognitive function [107].

A recent study revealed that after GCI in ovariectomized rats, remote ischemia postconditioning (RIP) effectively mitigated ischemic damage and improved cognitive function; enhanced the expression of prosurvival kinase pathways such as AKT, ERK1/2, and CREB; and suppressed CaMKIIα signaling in CA1 astrocytes induced by GCI. RIP was also found to alleviate reactive astrogliosis, which was accompanied by increased survival of A2 astrocytes and decreased death of A1 astrocytes, demonstrating that astrocytic E2 signaling is a critical target for the reparative effects of RIP following ischemic insult [62]. Moreover, in female forebrain neuronal aromatase knockout (FBN-ARO-KO) rats, a decrease in the A2 subtype of astrocytes and an increase in the A1 subtype were observed [59], which is regulated by ovariectomy after GCI; RNA sequencing revealed that astrocyte activation in FBN-ARO-KO mice was associated with the downregulation of the astrocyte-derived neurotrophic factors BDNF and IGF-1, as well as the astrocytic glutamate transporter [28]. These data provide novel evidence for a beneficial role of brain-derived E2 in astrocyte activation after injury.

### Astrocytic AROM in acute pain and chronic pain

AROM expression in the dorsal horn of the spinal cord suggests this local E2 could control spinal sensory processes including nociception [108]. In the spinal cord, AROM is expressed only in astrocytes and not in neurons [109, 110]. In an attempt to uncover the potential role of astrocyte AROM in formalin-induced acute pain and the mechanism by which spinal AROM activation is controlled, LET treatment improved late-phase formalin-induced nociceptive responses, suggesting that AROM activation (indicated by reduced phospho-serine levels) is involved in acute pain processing. Astrocytic sigma-1 receptor inhibition blocked the dephosphorylation of AROM and potentiated the pharmacological effect of LET on formalin-induced nociceptive responses, indicating that dephosphorylation is an important regulatory mechanism involved in the rapid activation of AROM and that spinal sigma-1 receptors mediate AROM dephosphorylation through an intrinsic calcineurin pathway [109].

Spinal cord astrocytes have also been shown to play a key role in the generation of cancer pain. Compared with saline-injected controls, fibrosarcoma cells produced robust mechanical hyperalgesia and cold allodynia in tumor-bearing mice; this increase was mirrored by significant increases in both spinal astrocyte activity and AROM expression in the dorsal horn. The administration of an AROM inhibitor reduced tumor-induced hyperalgesia in fibrosarcoma-bearing animals [110]. In the dorsal horns of a rat model of central pain syndrome, the levels of AROM, ERα and ERβ are increased after spinothalamic tract injury; LET treatment enhances mechanical allodynia in both hind paws and increases the number of astrocytes (as well as microglia), which are positively correlated with mechanical allodynia and gliosis in the dorsal horn [111].

### Astrocytic AROM in chemical injuries and sound wave stimuli

Both oxidative stress and nitrosative stress are complications of noncommunicable diseases, including endocrine and metabolic diseases and NDDs [112]. Nitrosative stress leads to protein nitrosylation, lipid peroxidation and DNA damage and therefore contributes to the neuronal dysfunction observed in the progression of NDDs and stroke [113–116]. In sheep, this phenomenon is closely related to the findings of Scrapie and Maedi Visna. In primary astrocyte cultures from 90-day-old fetal sheep brains, 3-nitro-L-tyrosine (a typical marker of nitrosative stress) treatment resulted in signs of deterioration and alterations in cell number and shape, whereas AROM expression was detected starting at 24 h and reached the highest levels at 72 h; nitrosative stress probably results in the local production of neuroprotective E2 by reactive astrocytes [117].

The organophosphate dimethoate (DMT) is an organophosphate pesticide that is extensively used for pest control, but its persistent residues raise concerns regarding human health [118]. A sublethal dose of DMT to rats resulted in hind limb disorders; impaired mitochondrial function; oxidative stress; DNA damage; and histological changes in the brain [119]. In cultured cortical astrocytes of male CD1 mice, DMT increased AROM, ER and cytokine levels, and the rescue effects of E2 were prevented by ER antagonists but imitated by ER agonists [120].

The song processing of song birds is modulated by the AROM-mediated rapid synthesis of local E2 when hearing conspecific song [121]. Based on finding that 528 Hz frequency sound wave, the 'miracle tone,' promotes tranquility and serve as an effective way for stress relief [122], Babayi et al. used primary astrocyte culture from adult male rats and found that sound waves with 528 Hz frequency in 100 dB intensity reduced AROM gene expression and total concentration of ROS in brain tissue, and the anxiety related behaviors were reduced, suggesting that sound wave reduced anxiety is related to decreased AROM in astrocyte [123].

### Sex differences associated neuroglial AROM functions

There are sex differences in the onset, prevalence, and outcome of numerous neurological disorders. One of the reasons for these sex differences may be the alterations in AROM expression in the CNS [124]. TBI induced an increase in AROM in astrocytes and the downregulation of neurotrophic factor receptors; however, when AROM activity was inhibited, worsened neurological deficits were specifically detected in females [103]. To investigate the mechanisms of sex-related differences in ischemic brain injury, Liu et al. first reported that astrocytic AROM activity and expression are greater in females than in males and that female astrocytes are more resistant to oxygen‒glucose deprivation (OGD) and oxidant cell death; similar results were also detected when female medium was transferred to cultured male cells [125]. When AROM was knocked out, this sex difference disappeared [26, 125]. In cultured OGD-induced astrocytes, female astrocytes are more resistant to OGD- and H_2_O_2-_induced oxidant death; AROM activity and expression are greater in female astrocytes than in male astrocytes, and an AROM inhibitor abolishes sex differences in OGD-induced cell death [125]. These findings were further confirmed in AROM-knockout (ArKO) mice, as WT female astrocytes were more resistant to OGD than were male astrocytes, and this sex difference was not detected in ArKO cells; cell death was significantly increased in ArKO females but not in astrocytes. Therefore, AROM appears to be essential for endogenous neuroprotection in females with traumatic and ischemic brain injury [26].

In an attempt to explore the mechanisms underlying sex differences in multiple sclerosis, local upregulation of AROM and ERβ in males but progesterone synthase and progesterone receptor in females was reported, indicating that these differences may contribute to sex differences in the prevalence and course of multiple sclerosis [82]. In cultured cortical astrocytes from male but not in those from female CD1 mice, DMT increased AROM, ERα, ERβ, steroidogenic acute regulatory, interleukin (IL)− 6, IL-1β, TNF-α, and IP10 mRNA levels. E2 administration decreased the mRNA levels of IL-6, IP10, TNF-α, and IL-1β in male but not in female cultures treated with DMT [120].

Palmitic acid (PA), the primary saturated fatty acid in the human diet, induces AROM expression mainly in females, and male astrocytes appear to be more susceptible to cellular damage when exposed to high concentrations of fatty acids. A high concentration of PA significantly reduced cell viability in male astrocytes and induced a greater increase in cytosolic ROS production in males, whereas female astrocytes presented higher superoxide ion levels in mitochondria. Female astrocytes treated with PA presented increased expression of antioxidant proteins, indicating a potential protective mechanism mediated by endogenous hormones [126].

### Astrocytic AROM regulatory mechanisms and cellular crosstalk

Understanding how glial AROM expression and activity are regulated by endogenous and exogenous stimuli is pivotal for novel therapeutic target development. There are multiple mechanisms controlling brain AROM activity at the genomic and non-genomic level as reviewed by an early report [127].

The upstream regulation of AROM expression is dynamically modulated by distinct signaling pathways under different pathological conditions, reflecting the complex interplay between cellular stress responses and neuroprotective mechanisms. Current studies show that brain AROM expression could be regulated by E2 treatment [91], N-methyl-d-aspartate/Ca^2+^ signaling cascade [37], CaV1.2-CaMKIIα signaling [62], calcineurin-mediated phospho-serine level of AROM [109], palmitic acid (PA) [126], naringenin [99], cytokines such as TNF, COX activity [82, 128–130] and IL-1 [49], innate immune system [131], endozepinergic signaling[132], 1,25-dihydroxyvitamin D3 [93], and growth factor such as VEGF [133]. Astrocytic AROM expression may be enhanced by traumatic brain injuries initiated cell death, edema, inflammatory mediators such as PGE2, NFκB, LL-1β, IL-6 and TNF-α [97, 134]. These findings highlight the diverse upstream mechanisms controlling glial AROM expression in response to CNS injuries. Furthermore, it is noteworthy that in breast cancer, AROM is regulated transcriptionally by hormones, cytokines, growth factors, non-protein coding RNAs and signaling molecules in a tissue specific manner as well as post-translationally by modifications such as phosphorylation, epigenetic regulation (histone acetylation, methylation, etc.) and Dishevelled protein [135], whether these also occur in neuroglial cells remains largely unclear. Targeting these pathways could provide novel therapeutic strategies to modulate AROM activity for neuroprotection while avoiding adverse effects.

At the cell–cell interaction level, it has been shown that forebrain neuronal AROM knockout (FBN-ARO-KO) rats showed decline in neural stem cells over 14-month age, increase in differentiation and over-activation of astrocytes and microglia as well as an increase in A1/A2 astrocyte subtype ratio at 18 month-old [59]. On the other hand, astrocytic AROM may function to regulated neuron and other glial cells, because it was shown that after GCI, both GFAP-ARO-KO and GFAP-ARO-iKO mice displayed increase in neuronal damage/loss through CaMKIIα-prosurvival pathway (AKT, ERK1/2 and CREB) cascade, microglia activation and then inflammation, and finally cognitive deficits [29, 30, 62]. Therefore, neuronal AROM loss affects neurogenesis, astrocyte differentiation and microglia activation, astrocytic AROM loss also affects neuronal survival and microglia activation, indicating their complex regulatory network.

Overall, the complex upstream regulation indicates astrocytic AROM is dynamically controlled by biochemical signals (E2, cytokines), biophysical stimuli (pressure), ischemic insults, and sex-specific factors. The JAK-STAT3 pathway is critical for driving protective A2 astrocyte polarization. The neuroprotective cellular crosstalk includes astrocyte-derived E2 inhibits pro-inflammatory microglial activation, E2 enhances neuronal survival and synaptic plasticity via genomic (ERα/ERβ) and nongenomic (GPR30) pathways and self-regulation reduces harmful reactive astrogliosis and promotes reparative A2 astrocytes. The therapeutic significance suggests targeting astrocytic AROM or downstream effectors may enhance recovery post-injury, and sexual dimorphism necessitates sex-specific dosing strategies for AROM-targeted therapies.

**Fig. 2:**
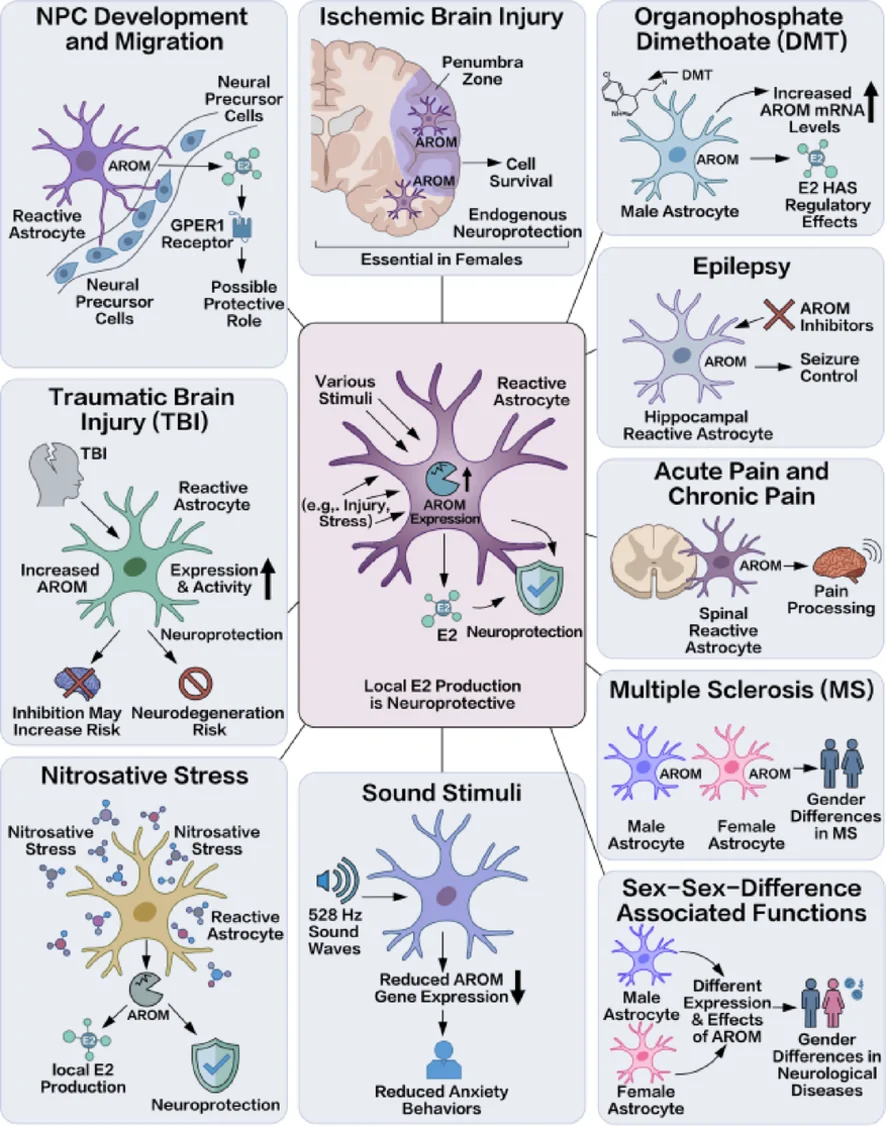
The functions, mechanisms, and sex differences of neuroglial AROM. These injury-, stimulus- and disease-related functions are mostly based on findings from astrocytic AROM expression and regulation

The above findings are summarized in Table 2 and Fig. 2.

**Table 2:** Targeting glial AROM for neuroprotection: expression and mechanisms in CNS injury models

Category	Specific mechanism/pathway	Interaction/effect	Functional outcome	Key references
Upstream regulation	E2 feedback signaling	Autocrine regulation of astrocytic AROM expression	Maintains neuroprotective E2 synthesis	[91]
	Cytokine signaling (e.g., TNF, COX, IL-1), innate immune	Dose-dependent inhibition of AROM in neonatal cortical astrocytes	Modulates injury-induced AROM activity	[49, 82, 128–131]
	NMDA/Ca^2+^ signaling	Positive regulates AROM	Alteration in estradiol	[37]
	CaV1.2-CaMKIIα	Negative regulates AROM	Alteration in estradiol	[62]
	Mechanical pressure (TBI)	↑ AROM mRNA/protein in primary cortical astrocytes after pressure exposure (25 mm Hg)	Adaptive response to traumatic injury	[102]
	Ischemia (GCI/MCAO)	↑ Astrocytic AROM in penumbra/peri-infarct zones	Promotes survival of ischemic neurons	[105, 106]
	JAK-STAT3 pathway	Downregulated in GFAP-ARO-KO mice after GCI; critical for A2 astrocyte gene expression	Essential for neuroprotection and anti-inflammatory response	[29]
	Sigma-1 receptor & calcineurin	Dephosphorylation of AROM (e.g., in pain models)	Mediates acute nociceptive responses	[109]
	Sexual dimorphism	Higher basal/induced AROM activity in females after TBI/ischemia	Greater female resilience to brain injury	[103]
Cellular interactions	Astrocyte → Microglia	Astrocyte-derived E2 ↓ microglial activation via ERs/GPR30—CaMKIIα pathway	Suppresses neuroinflammation	[29, 30, 62]
	Autocrine effect on astrocytes	E2 signaling ↓ reactive astrogliosis; ↑ A2 neuroprotective subtype	Reduces glial scar formation; enhances repair	[59, 62]
	Astrocyte → Neurons	E2 activates neuronal ERα/ERβ/GPR30 → ↑ neurogenesis, synaptic protein expression	Improves cognitive function & neuronal survival	[107]
	Astrocyte → Neural precursors	AROM^+^ astrocytes protect migrating neuroblasts in V-SVZ via GPR30	Maintains actin cytoskeletal homeostasis in neuroblasts	[78]
	Astrocyte → Neuron survival	RIP ↑→ astrocytic E2 → ↑ AKT/ERK/CREB pathways; ↓ CaMKIIα	Mitigates ischemic neuronal damage	[62]

↑: increase or upregulation; ↓: decrease or downregulation

## Induced astrocytic AROM expression and function in song birds

**Fig. 3:**
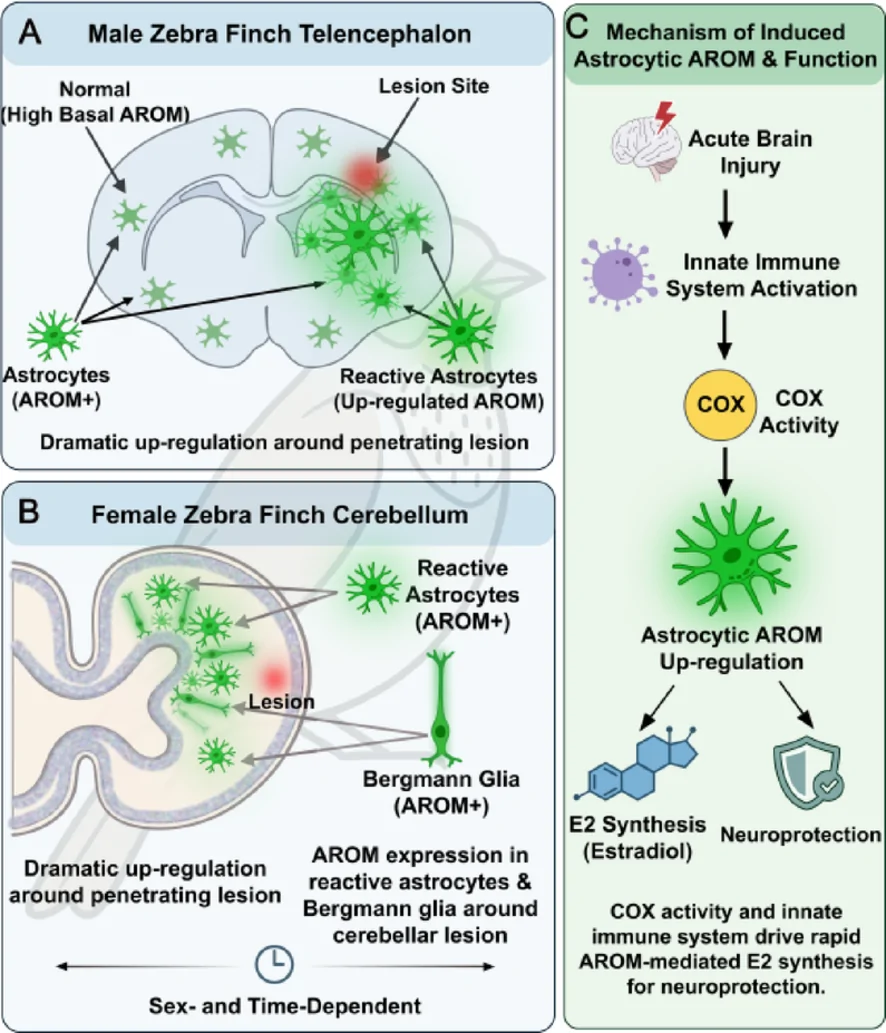
Induced astrocytic AROM expression and function in songbirds after neural injury. In general, songbird brains exhibit high levels of AROM, which can be regulated by injuries such as those in rodents, and this upregulation is also neuroprotective. A: Penetration injury induces astrocytic AROM around lesions in males. B. Cerebellar lesions induce AROM in astrocytes and Bergmann cells in females. C. Role of COX in acute brain injury-induced upregulation of astrocytic AROM in song birds

Songbirds are unique in that AROM is expressed at unusually high levels in the telencephalon of both males and females. AROM expression has been reported in sensory and integrating nuclei of the hindbrain in Japanese quail [136]. In adult male zebra finches, penetrating neural injury dramatically upregulated AROM expression in radial glial cells lining the lateral ventricle adjacent to the lesioned hippocampus, along with a dramatic accumulation of newly generated, BrdU-labeled cells along AROM-positive fibers, indicating that radial aromatization may be a key feature in the repair of the vertebrate brain following neural injury (Fig. 3A) [60, 137, 138]. Additionally, cerebellar lesions of adult female zebra finches produced deficits in motor and cognitive aspects of a spatial task, and lesions induced the expression of AROM in reactive astrocytes and Bergmann glia (specialized cerebellar glia derived from radial glia but sharing the characteristic features of mature astrocytes [139]) around the lesion, indicating that glial AROM may improve the recovery of cognitive function after injury [140]. Furthermore, in adult male and female zebra finches, unilateral penetrating brain injury induced AROM and E2 in the injured hemisphere but not in the uninjured hemisphere, without affecting the expression of ER mRNA but significantly decreased the level of androgen receptor mRNA in the injured lobe relative to that in the contralateral uninjured hemisphere (Fig. 3B) [141]. Thus, injury-induced upregulation of astrocytic AROM may be a general characteristic of the injured vertebrate brain, indicating the conservation in evolution.

The above induction may be sex- and time dependent, since brain injury induces more rapid AROM in reactive astrocytes in females than in males. Cyclooxygenase (COX) activity is necessary for injury-induced reactive astrocytic AROM expression around the site of damage, and increased central E2 lowers neuroinflammation. Indomethacin (a COX inhibitor) reduces AROM expression and E2 content at 6 h but not 24 h following injury in females; however, in males, this inhibition is apparent at 24 but not 6 h after treatment. This acute brain injury-induced increase in E2 is neuroprotective, with established influences on apoptosis, gliosis, cytogenesis, neurogenesis and neuroinflammation; decreases in secondary damage; and improved recovery [128, 129, 142, 143], as indicated in Fig. 3C.

## Conclusions and prospective

Previous studies have reported that neuronal AROM-initiated estrogen signaling plays important roles in the central regulation of nonreproductive functions, such as learning and memory and energy metabolism [59, 65, 68, 69, 144–146]. While neurons maintain the highest constitutive AROM levels, recent studies have shown that AROM is expressed in neuroglial cell populations, including astrocytes, microglia, oligodendrocytes, and ependymal cells. AROM expression profiles in glial cells exhibit dynamic, context-dependent induction, particularly in response to neurodegenerative conditions, brain injury, and ischemia. Among all nonneuronal cells, astrocytic AROM is the most significantly upregulated following TBI and ischemic stroke, contributing to neuroprotection via both genomic ERα/ERβ and nongenomic GPR30 signaling pathways. Moreover, sexual dimorphism is a key feature, with females displaying higher basal and injury-induced AROM activity, which correlates with their greater resilience to ischemic damage. Moreover, spinal AROM in astrocytes may play a role in multiple sclerosis and chronic and acute pain processing. In addition to astrocytes, microglial AROM exhibits sex-specific regulation under inflammatory conditions, whereas oligodendrocyte and ependymal AROM may influence myelination and cerebrospinal fluid dynamics, respectively.

Investigation the cell- and mechanism- specific AROM expression pattern in ageing, neural disease and brain injuries is beneficial for the prevention and treatment of these neural disorders. Despite the advances mentioned above, challenges remain in translating these findings into therapies, particularly regarding blood–brain barrier penetration and sex-specific dosing regimens. Current AROM inhibitors were predominantly employed in the treatment of hormone receptor-positive, HER2-negative advanced breast cancer (HR^+^HER2^−^ ABC). While randomized controlled trial (RCT) revealed that in these patients, AROM inhibitors induced cognitive function decline in a small group of patients [147], clinical trial showed that AROM inhibitor treatment did not alter cognitive function, but significantly enhanced depressive symptoms and sleep disturbances in these patients [148] and in peripubertal boys with idiopathic short stature [149]. These trials provide potential clues for the enhancement, not inhibition, of endogenous AROM activity and expression after brain injury and normal ageing, based on the upstream regulatory mechanisms as mentioned. RCT/clinical trials regarding AAV-mediated, brain-blood-barrier penetrable, cell-type specific AROM over-expression vectors, and small molecules that activate AROM expression, should be explored.

Disease-associated cell sub-types, including astrocytes, microglia and oligodendrocytes with single-cell RNA-sequencing, have been identified in AD and other brain disorders and show promising therapeutic potential [150–156]. The single-cell omics, together with spatial transcriptomics/proteomics, could be used to delineate AROM-expressing glial subpopulations and their functional heterogeneity, alongside the construction of conditional knockout models to dissect cell type-specific contributions. Therapeutic strategies targeting astrocytic AROM or downstream effectors hold promise but require rigorous preclinical validation to optimize efficacy. Additionally, exploring the roles of microglial and oligodendrocytic AROM in neuroinflammation and remyelination could reveal novel mechanisms for treating multiple sclerosis and other demyelinating disorders. Furthermore, clinical studies should also investigate AROM polymorphisms and their associations with disease susceptibility (for example, with AD [72], depression [157, 158], and astrocytoma [159]), whereas advanced imaging and organoid models may provide deeper insights into human-specific regulation. Ultimately, a comprehensive understanding of neuroglial AROM could pave the way for precision therapies for stroke, TBI, neurodegeneration, and other CNS disorders, bridging the gap between fundamental research and clinical application.

## Data Availability

No datasets were generated or analysed during the current study.

## Abbreviations

ABI: Acquired brain injury
AD: Alzheimer’s disease
ALS: Amyotrophic lateral sclerosis
ArKO: AROM-knockout
AROM: Aromatase
CCI: Controlled cortical impact
CDV: Canine distemper virus
CNS: Central nervous system
COX: Cyclooxygenase
DMT: Dimethoate (organophosphate pesticide)
E2: Estrogen (17β-estradiol)
EAE: Experimental autoimmune encephalomyelitis (MS model)
ER: Estrogen receptor
FBN-ARO-KO: Forebrain neuronal aromatase knockout
FISH: Fluorescent in situ hybridization
GCI: Global cerebral ischemia
GFAP: Glial fibrillary acidic protein
GFAP-ARO-iKO: Inducible astrocyte-specific AROM knockout
GFAP-ARO-KO: GFAP promoter-driven aromatase knockout
GPR30/GPER1: Membrane estrogen receptor
IL: Interleukin
IP10: Interferon-c-inducible protein 10
KA: Kainic acid
LET: Letrozole (AROM inhibitor)
MCAO: Middle cerebral artery occlusion
MS: Multiple sclerosis
NDDs: Neurodegenerative diseases
NFκB: Nuclear factor Kappa B
OGD: Oxygen‒glucose deprivation
PA: Palmitic acid
PD: Parkinson’s disease
PFC: Prefrontal cortex
PGE2: Prostaglandin E2
PPT: ERα agonist
RMS: Rostral cerebral artery occlusion

## Declarations

### Statement

The figures were originally generated with Nano Banana Pro. The authors reviewed and edited thecontent as needed and take full responsibility for the content of the publication.
